# Functional abnormalities in the cortical processing of sound complexity and musical consonance in schizophrenia: evidence from an evoked potential study

**DOI:** 10.1186/1471-244X-13-158

**Published:** 2013-05-30

**Authors:** Kuan-Yi Wu, Ching-Wen Chao, Ching-I Hung, Wei-Hong Chen, Yung-Ting Chen, Sheng-Fu Liang

**Affiliations:** 1 Department of Psychiatry, Chang Gung Memorial Hospital at Linkou & College of Medicine, Chang Gung University, Taoyuan, Taiwan; 2 Department of Music, National Taiwan Normal University, Taipei, Taiwan; 3 Department of Computer Science and Information Engineering & Institute of Medical Informatics, National Cheng Kung University, Tainan, Taiwan

**Keywords:** Music perception, Auditory evoked potential, Event-related potential, Schizophrenia

## Abstract

**Background:**

Previous studies have demonstrated functional and structural temporal lobe abnormalities located close to the auditory cortical regions in schizophrenia. The goal of this study was to determine whether functional abnormalities exist in the cortical processing of musical sound in schizophrenia.

**Methods:**

Twelve schizophrenic patients and twelve age- and sex-matched healthy controls were recruited, and participants listened to a random sequence of two kinds of sonic entities, intervals (tritones and perfect fifths) and chords (atonal chords, diminished chords, and major triads), of varying degrees of complexity and consonance. The perception of musical sound was investigated by the auditory evoked potentials technique.

**Results:**

Our results showed that schizophrenic patients exhibited significant reductions in the amplitudes of the N1 and P2 components elicited by musical stimuli, to which consonant sounds contributed more significantly than dissonant sounds. Schizophrenic patients could not perceive the dissimilarity between interval and chord stimuli based on the evoked potentials responses as compared with the healthy controls.

**Conclusion:**

This study provided electrophysiological evidence of functional abnormalities in the cortical processing of sound complexity and music consonance in schizophrenia. The preliminary findings warrant further investigations for the underlying mechanisms.

## Background

Schizophrenia is a catastrophic psychiatric disorder that is characterized by positive (hallucinations or delusions) and negative symptoms (apathy or anhedonia), cognitive impairment, and mood or anxiety symptoms. It is commonly associated with impairments in social and occupational functioning. The characteristics of psychopathology in schizophrenia are suggestive of functional and structural temporal lobe abnormalities [1-4], some of which have been found to be located in the superior temporal gyrus, close to the auditory cortical regions of the brain [3-6]. Considering the interaction between temporal lobe abnormalities and auditory neurophysiology in schizophrenia [3,7], it is intriguing to explore whether the central processing of musical sound in patients with schizophrenia differs from that in healthy people.

A number of researchers have demonstrated abnormal auditory processing in schizophrenia by evoked potential studies. The most replicated findings include a reduction in the amplitude of the P3 component (P300) [8,9], failure to inhibit the second response to paired-click stimuli (p50) [10,11] and a gating deficit in N1(N100) and P2 component (P200) [12-14]; however, most prior research was limited by a reliance on simple oddball tasks, which requested that subjects detect auditory targets in a string of pure tonal stimuli [7]. These abnormal auditory processing results cannot demonstrate deficits in the perceptive processing of musical sound [3]. Therefore, the phenomenon of cortical processing of musical sound in schizophrenia, which is close to the real-world experience of music, remains poorly understood, and has been discussed only rarely in related research to date.

One major reason for which everyone can perceive music as being full of variety is that musical sounds consist of more than one pitch, and combinations of various pitches are used to construct varying degrees of complexity and consonance. These degrees can be perceived due to the related frequency ratios: the simpler ratios, the more consonant. Therefore, the complexity and consonance of sound are key features in experiencing music [15]. Use of the auditory evoked potentials (AEPs) technique has proved a powerful tool for comparing music processing in the brain [16,17].

The present study explored this interesting phenomenon through analysis of responses to the features of sound complexity and music consonance. The musical sounds used in this study as the acoustic stimuli consisted of two kinds of newly-created sonic entities, (1) intervals (dyads, combinations of two pitches) and (2) chords (triads, combinations of three pitches), of varying degrees of consonance. The aim of this study was to investigate whether deficits in the central perception of musical sounds are present in patients with schizophrenia.

## Methods

### Subjects

Twelve medicated day-hospital patients with schizophrenia from the Department of Psychiatry of Chang Gung Memorial Hospital and twelve age- and sex-matched healthy controls were recruited to this study. All subjects were right-handed, reported normal hearing, and had not received any formal music training. Each of the two groups comprised 8 females and 4 males who ranged in age from 20 to 29 years, with a mean (SD) age of 24.7 (2.8) years. Schizophrenia diagnoses were confirmed according to the DSM-IV criteria on the basis of a clinical interview and a review of the case files, and degrees of psychopathological symptoms were evaluated by a semi-structured interview using the Positive and Negative Syndrome Scale (PANSS) [18]. Control-group participants were excluded if they had a current or past history of any psychiatric illness based on screening using the Mini-International Neuropsychiatric Interview (MINI) [19]. All participants were also screened to exclude those with a history of seizure, other neurological insult or illness, or a history of substance abuse. The patient group consisted of patients with schizophrenia with a mean duration of illness (SD) of 6.1 (2.5) years prior to testing. The mean (SD) PANSS score in the patient group was 59.5 (8.0), and the mean (SD) scores in the three subscales, the Positive, Negative and General Psychopathology scales, were 13.6 (2.4), 16.1 (3.7) and 29.8 (5.1), respectively. At the time of testing, all schizophrenic patients were receiving treatment with atypical antipsychotic medication; two were being treated with Olanzapine 15 mg/d, two with Clozapine (mean dosage 375 mg/d, 200 and 550 mg/d), three with oral Risperidone (mean dosage 4 mg/d, range 2–6 mg/d), four by Risperidone intramuscular injection (mean dosage 43.75 mg, range 37.5–50 mg every two weeks) and one with Zotepine (400 mg/d). One of the two patients being treated with Clozapine was also receiving combination therapy consisting of Sulpride (100 mg/d); one of the three being treated with oral Risperidone was receiving Haloperidone (7.5 mg/d) in combination and another a 20 mg Flupenthixol decanoate injection every week; two patients were taking mood stabilizers (Lithium, Sodium valproate) and another two were taking an antidepressant (Fluoxetine). This study was approved by the Institutional Review Board of Chang Gung Memorial Hospital. All subjects had given written informed consent for the procedures to be carried out before AEP testing.

### Acoustic stimuli and experimental design

The stimuli consisted of two types of sonic structure, intervals (2 tones) and chords (3 tones), created with sinusoidal tones. The tones (350 ms in duration with a 100 ms fade-out time), tuned to the equal-tempered chromatic scale in the range of G# (104 Hz) to E5 (659 Hz), 16 bit, 44.1 kHz, were first created at a fixed amplitude.

**Figure 1:**
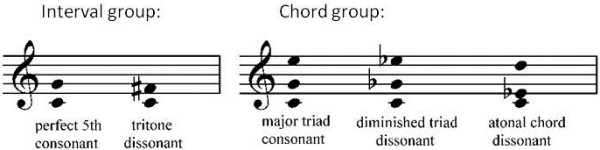
The auditory stimuli consisting of 2 types of interval and 3 types of chord, of which 12 different transpositions were evenly distributed within the range of G# (104 Hz) to E5 (659 Hz) - a total of 60 different-pitched intervals/chords.

In the interval group, tones were paired at pitch intervals of 6 (tritone) and 7 (perfect fifth) semitones to produce 24 different dyads, 12 dyads per kind. The perfect fifth (e.g., 220:330hz) is considered more consonant than the tritone (e.g., 220:311hz) because its frequencies are related by simpler ratios. In the chord group, 3 tones were chosen to construct major triads (consonance), diminished triads (dissonance), and atonal chords (lack of a tonal center) to generate 36 different chords, 12 per kind. The major triad is considered more consonant than the diminished triad or atonal chord because it comprises tones with fundamentals that are related by simple frequency ratios. Each of 5 kinds of sonorities (intervals/chords) was constructed so that the constituent simple tones of the 12 dyads/chords were evenly distributed within the above-defined frequency range, which ensured that the overall frequency characteristics were comparable under all conditions (Figure 1).

In each session, the stimuli (120 trials; a total of 60 different-pitched dyads/chords, each appearing twice) were presented randomly with an inter-stimulus interval (ISI) randomized in the range of 2–4 seconds to minimize the effect of expectancy. Each session was performed 5 times (a total of 600 trials) for each subject with a short rest in-between. Professional earphones (Audio-Technica Ath-Pro5) were utilized for binaural stimulus presentation and the intensity was controlled at 65 dB. Subjects were instructed to listen attentively to the acoustic stimuli with closed eyes.

### EEG recording

**Figure 2:**
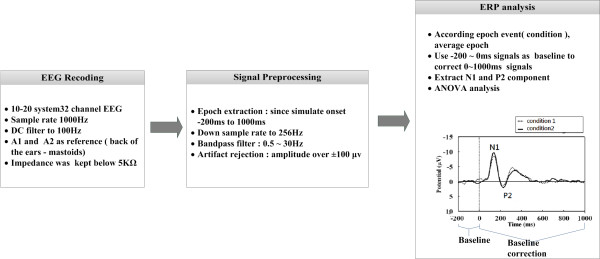
EEG recording and signal processing.

EEG recording and signal processing were shown in Figure 2. EEGs were continuously recorded in DC mode at a sampling rate of 1000Hz using 32 electrodes mounted in elastic caps and referenced to A1–A2, in accordance with the 10–20 international system. The impedance at each scalp electrode was kept below 5KΩ. The electrode positions, physical landmarks, and head shape were digitized using a Polhemus Fastrak digitizer and the 3D spaceDx software in the Neuroscan SCAN package. The EEG was amplified using a Neuroscan NuAmp and the filter setting was DC to 100Hz, 6 dB/octave attenuation. The digitized EEG data were passed through a digital band-pass filter of 0.5–30 Hz (EEGLAB, FIR filter) to eliminate slow drifts and muscular artifacts.

EEG data from 200 ms prior to and 1000 ms following the onset of each stimuli were segmented. To reduce the differences of component amplitudes in computation and prevent the baseline drift, -200 ~ 0 ms signals were used as baseline to correct 0 ~ 1000 ms signals in each epoch. A typical adult human EEG signals are about 10 μV to 100 μV in amplitude when measured from the scalp [20]. Based on EEG signal standardized processing method [21], we set 100 μv as the rejection level to detect eye artifacts (including eye blinks, eye movements and extra-ocular muscle activity) and removed the trials which exceed that level. After the removal, there were 5467 trails preserved in the healthy controls, 5729 trails in the schizophrenic patients, both over 70% of the total number of 7500 trials. Generally, N1 and P2 represent the first large negative amplitude and second large positive amplitude after onset of stimulus. In this study we found N1 and P2 latency were located in 100–150 ms and 180– 250 ms; therefore, peak amplitudes of the N1 and P2 components were determined as the peak reversals during the time intervals of 100–150 ms and 180–250 ms after stimulus onset, respectively. Topographic maps were generated using EEGLAB to define the spatial distributions and dynamics of the activity on the scalp surface [22].

### Statistical analysis

Regarding sound complexity, the amplitudes and latencies of AEPs elicited by the interval and cord stimuli were analyzed using repeated measures analysis of variance (ANOVA) and were compared between groups (patient and control). Regarding music consonance, repeated-measures ANOVA was conducted to investigate the modulation of potentials evoked by acoustic stimuli of varying degrees of consonance consisting of chords (atonal chords, diminished chords, and major triads) and intervals (tritones and perfect fifths). Pearson correlations were performed to examine the relationship between the amplitudes of the AEPs and the psychopathological ratings. Significant correlations were validated using non-parametric Spearman rank-order correlations. In all analyses, statistical significance was set at the level of *p* < 0.05.

## Results

Components N1 and P2 were observed within several hundred milliseconds of stimulus presentation. The latency range and topographic distribution did not differ between the schizophrenic patients and the healthy controls. Based on topographical analysis, the N1 and P2 amplitudes presented the largest values at the frontocentral sites and appeared equally present at electrode sites over each hemisphere. Repeated-measures ANOVA was performed for the anterior (Fz electrode), central (Cz), and posterior (Pz) sites to analyze the effects of chord and interval stimuli on the amplitudes of the N1 and P2 components.

### Sound complexity

**Figure 3:**
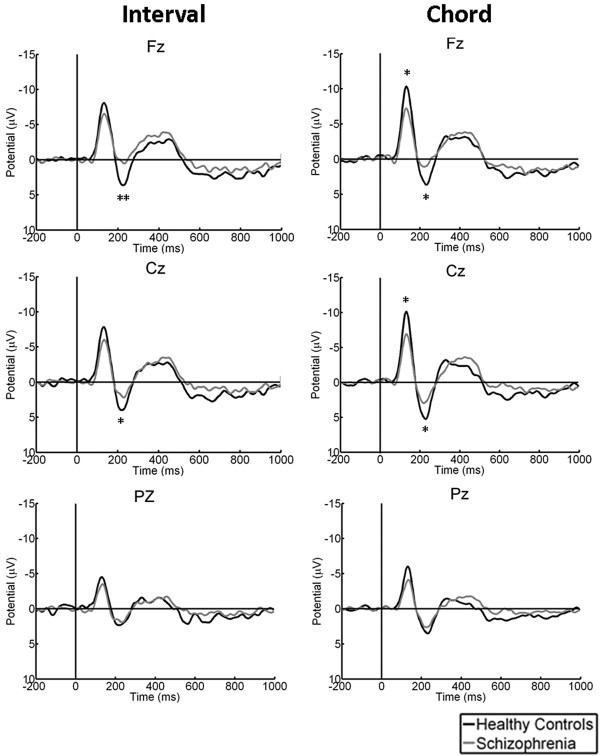
**Between-group (healthy controls and schizophrenic patients) comparison of AEPs elicited by interval (left) and chord (right) stimuli at different regions (anterior, top; central, middle; posterior, bottom).** * *p* <0.05, ** *p* < 0.001.

**Figure 4:**
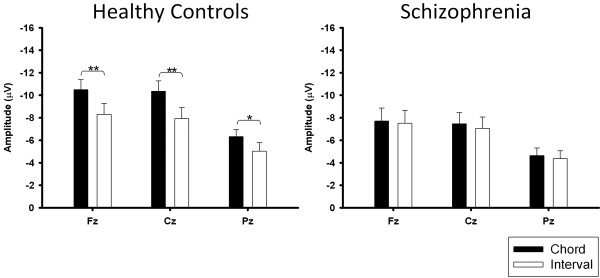
**N1 amplitude elicited by interval and chord stimuli in the healthy control (*n*** **= 12) and schizophrenia groups (*n*** **= 12).** * *p <* 0.05, ** p < 0.001.

Significant reductions in the amplitudes of the N1 and P2 components elicited by both chord and interval stimuli were observed in the schizophrenic patients (Figure 3). In addition, the N1 amplitudes elicited by chords were found to be significantly greater than those elicited by intervals in the control group; however, this phenomenon was not observed in the schizophrenic patients (Figure 4).

### Music consonance

**Figure 5:**
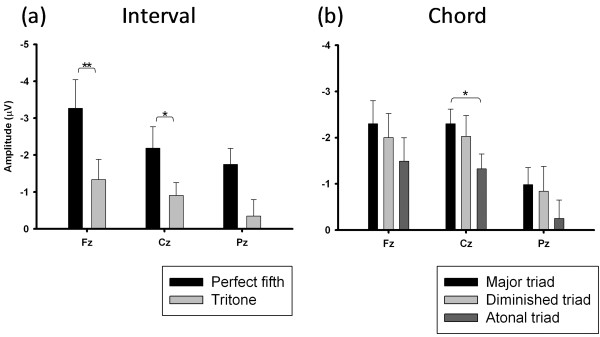
**Differences in reduction of amplitude of P2 evoked by (a) intervals and (b) chords in schizophrenic patients.** * *p* < 0.05, ** *p* < 0.001.

Schizophrenic patients showed reduced N1 and P2 components elicited by both chord and interval stimuli. We further conducted separate analyses of reductions in amplitudes for different intervals (perfect fifth and tritone) and chords (major triads, diminished triads and atonal chords) respectively. The results showed schizophrenic patients had greater P2 reductions elicited by perfect fifth than tritons. The reduction in the N1 and P2 amplitudes elicited by chords was observed to be the greatest in major triads and the lowest in atonal triads. The significant differences in the reduction of the P2 amplitude elicited by the various intervals and chords in the schizophrenic patients are shown in Figure 5.

Relationship between the amplitudes of N1 and P2 and measures of psychopathological severity in the schizophrenic patients.

The N1 amplitude elicited by both interval and chord stimuli was not significantly correlated with the total PANSS score (the range of correlation coefficients 0.21 to 0.48) or the scores of the three subscales (positive subscale −0.43 to −0.05; negative subscale −0.44 to 0.03; somatic subscale 0.003 to 0.22); similarly, correlations between the P2 amplitude and PANSS scores remained statistically non-significant (total score −0.15 to 0.40; positive subscale −0.27 to 0.23; negative subscale 0.13 to 0.56; somatic subscale −0.24 to 0.18). These results were all validated using the non-significant Spearman’s rank correlation coefficient.

## Discussion

Our results showed that the N1 and P2 amplitudes were reduced in the schizophrenic patients as compared with the healthy controls. The reduced N1 component has been replicated in a large number of previous studies [23-25], but the P2 response is relatively understudied [26,27]. Most studies have relied mainly on standard oddball tasks or gating tasks, which differ from the listening tasks used in this study. The N1 component represents the primary stimulus-dependent response [28,29]. The P2 component represents subsequent potentials, which may reflect the mental operation of perception within processing-associated brain regions [28]. Another important finding was that when the healthy controls were subjected to interval and chord stimuli, an obvious difference in the N1 component elicited by these two different musical objects was discovered; however, this difference was not observed in the schizophrenic patients. It meant that the patients appeared unable to respond to the subtle dissimilarity between intervals and chords in this study. This finding might partly imply deficits in sound complexity perception in schizophrenia.

With regards to comparison of the perception of music consonance between patients and controls, it was observed that consonant intervals/chords contributed more highly to the discrepancy in the AEP amplitudes than dissonant ones; in other words, healthy controls can perceive consonant musical sounds to a greater degree than schizophrenic patients can. This result might suggest deficits in music consonance perception in schizophrenia. To our knowledge, this study was the first to investigate aspects of the central processing of sound complexity and music consonance in schizophrenia.

An interesting finding of this study was that psychopathological severity had no significant correlation with N1/P2 amplitude in the schizophrenic patients. Some previous studies reported modest associations [30,31] but most revealed no correlation based on standard oddball experiments [24,27,29,32-36]. This study is in line with the assumption that the reduced N1/P2 amplitudes might represent primarily a trait marker of schizophrenia.

The statistical analyses in this study using univariate ANOVA may not be optimal. We conducted a three way ANOVA to rework the statistical analyses. Repeated-measures ANOVA were used to evaluate the group effects with group (patients/controls) as a between-subjects factor, and stimulus type (perfect fifth, tritone, major triads, diminished triads and atonal chords) and region (each EEG channel) as within-subjects factors. The results showed there were significant group by region interactions corresponding to P2 for each music stimulus: perfect fifth (F(1,26) = 4.54, p < 0.001), tritone (F(1,26) = 2.504, p < 0.001), major triads (F(1,26) = 4.72, p < 0.001), diminished triads (F(1,26) = 1.96, p = 0.003) and atonal chords (F(1,26) = 2.37, p < 0.001). Further post-hoc analyses showed the number of EEG channels which could detect significant differences between the patients and controls were observed to be the greatest in major triads and the lowest in atonal triads. These findings demonstrated that the consonant sounds may cause more numbers of EEG channel to detect differences between the two groups than the dissonant ones. Overall, these findings were consistent with the results previously reported using univariate ANOVA and did not influence the conclusion.

Interpretation and generalization of the current findings must acknowledge several limitations of this study. First, medication effects were a major limitation. However, a critical review study indicated that studies revealing a reduced N1 component to be a direct consequence of antipsychotics treatment are rare [29]. Furthermore, the association between N1/P2 components and clinical improvement under medication is also weak [34,37,38]. The recruited patients in previous studies were of medicated patients. Thus, medication effects still remained as inevitable confounding factors. Second, this study recruited schizophrenic patients of a young age with an early onset and chronic duration of illness, and therefore the results cannot be generalized to other patient samples. Third, the N1 and P2 components cannot be referred to as a single cortical process [29]. These AEP components might reflect information processing of multiple domains of higher cortical functions in the brain, including, but not limited to, perception, attention, memory and executive function. Future investigation may consider the use of musical excerpts from real repertoires to gain further insight into the functional and structural deficits underlying the pathophysiology of musical sound perception in schizophrenia.

## Conclusions

Schizophrenic patients exhibited significantly lesser activation of the N1 and P2 components than healthy controls while perceiving musical sounds as acoustic stimuli. This study provided electrophysiological evidence that schizophrenic patients might suffer deficits in the cortical perception of sound complexity and music consonance. The underlying mechanisms warrant further investigations.

## Competing interests

The authors declare that they have no competing interests.

## Authors’ contributions

KYW, CWC and SFL conceived the trial. KYW, CWC and SFL designed the detailed protocol. KYW, CWC, CIH and WHC performed the experiments. KYW, WHC, YTC and SFL analyzed the data. KYW, CWC and SFL wrote the first draft of the paper. All authors contributed to development and revision of the manuscript. All authors have read and approved the final manuscript.

## Pre-publication history

The pre-publication history for this paper can be accessed here:

http://www.biomedcentral.com/1471-244X/13/158/prepub
